# Supplementary material for: The genomic epidemiology of shigellosis in South Africa
Source: Nat Commun. 2023 Nov 24;14:7715. doi: 10.1038/s41467-023-43345-5 (PMC10673971; doi:10.1038/s41467-023-43345-5)
Supplement: Supplementary file 1 — Supplementary Information [file 41467_2023_43345_MOESM1_ESM.pdf]

Supplementary items

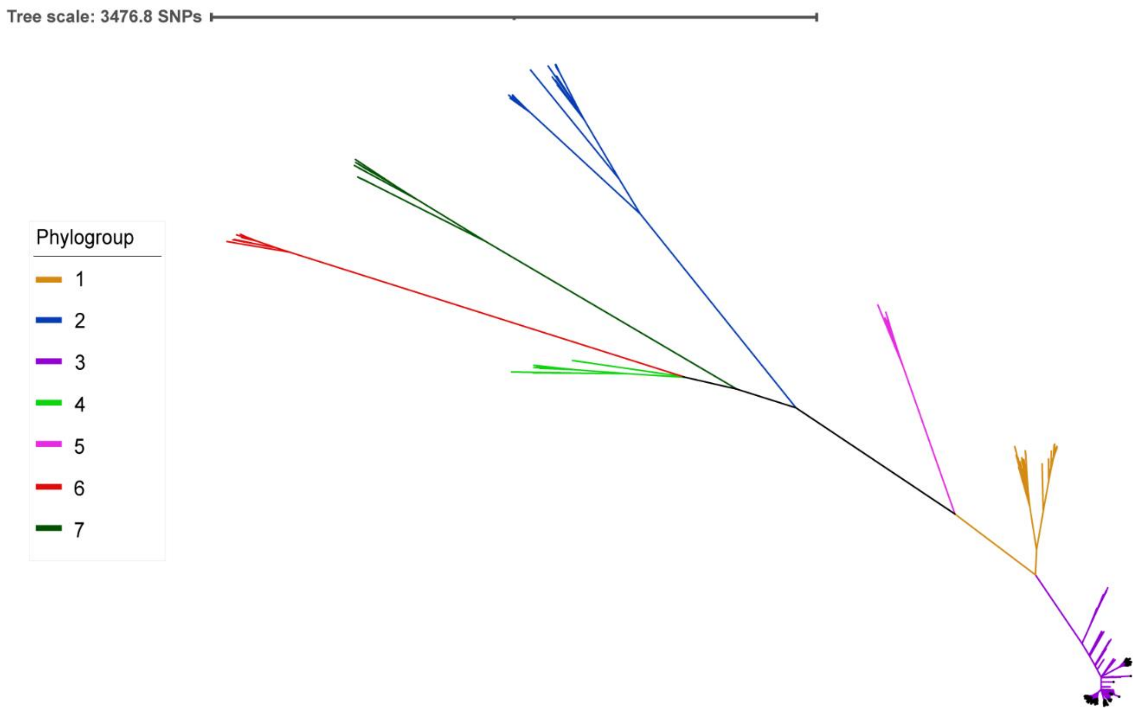

Supplementary figure 1. Maximum likelihood phylogeny of *S. flexneri* study isolates and global representative isolates from across the known global phylogeny.

Study isolates are indicated by black terminal node dots, all within Phylogroup 3. Phylogroup branches are coloured according to inlaid key.

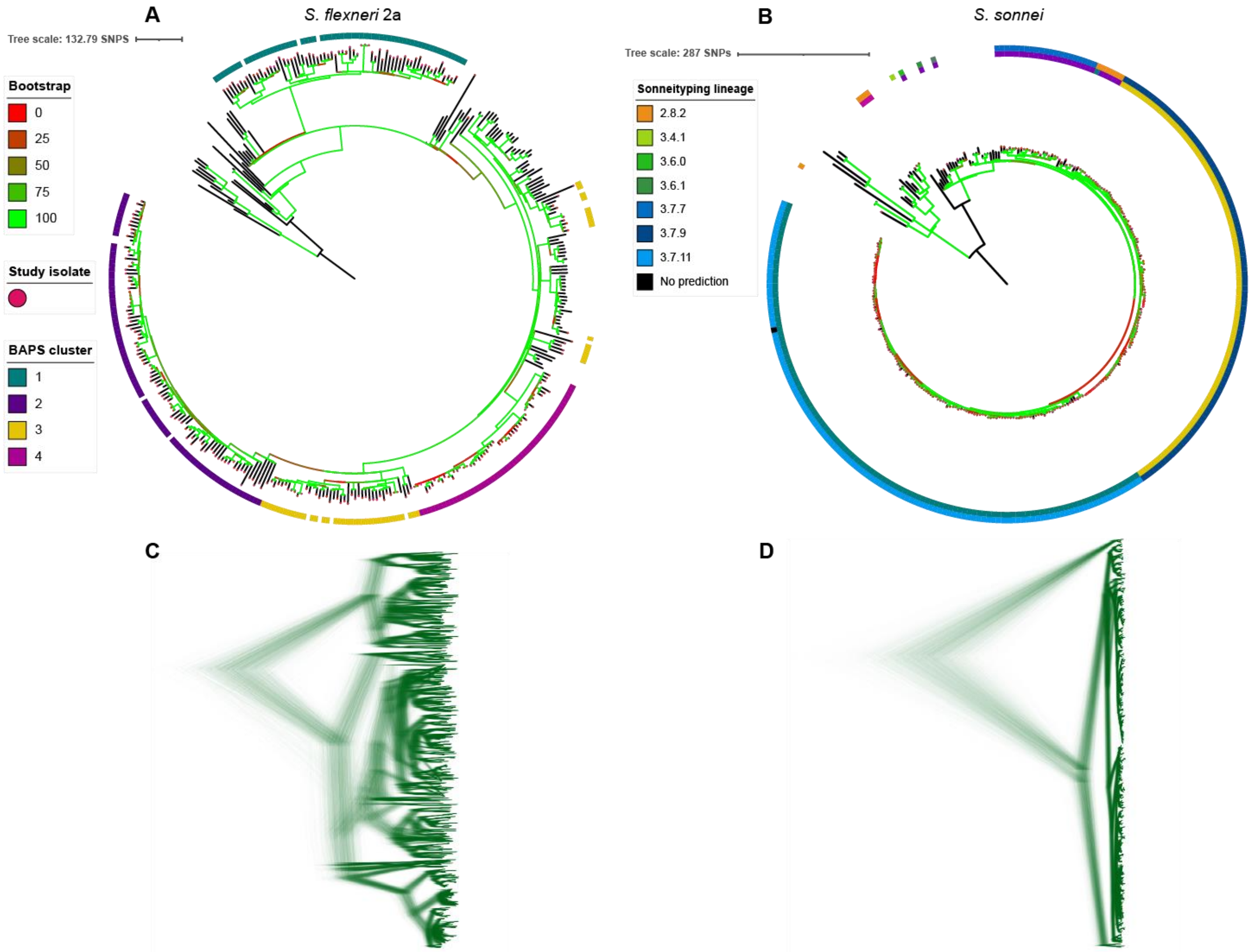

Supplementary figure 2. Support for South Africa Shigella phylogenetic trees.

A) Bootstrap support for the Phylogroup 3 *S. flexneri* 2a phylogeny (left) and the *S. sonnei* phylogeny (right).

B) Support for the Bayesian phylogenies for *S. flexneri* 2a (left) and S. sonnei (right), visualised in densitree.

Source data are provided as a Source Data file.

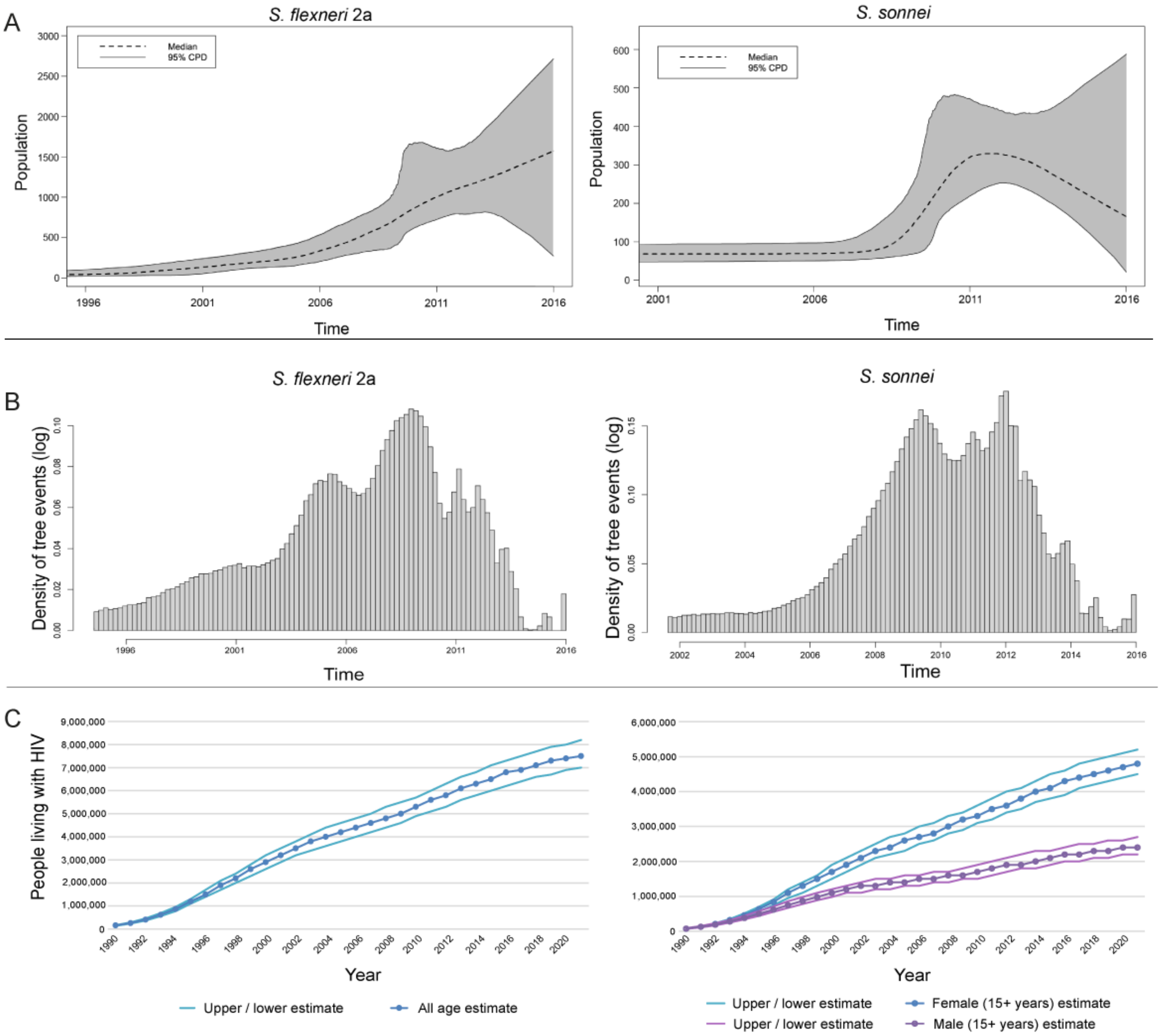

Supplementary figure 3. Population dynamics of South Africa *Shigella* compared to number of people living with HIV in South Africa.

Estimated population dynamics (A) (images reflected to invert time scales) of *S. flexneri* 2a (left) and *S. sonnei* (right). Dotted line shows the median estimated population size through time, grey shaded area shows 95% HPD estimated population size estimate. B) Number of tree coalescent events through time, for *S. flexneri* 2a (left) and *S. sonnei* (right). The number of the coalescent events dropped off into the past, meaning the results are, consequently, less reliable. C) Number of people living with HIV in South Africa, data from AIDSinfo.UNaids.org; all ages (left) and 15+ years old (right) split by sex. Source data are provided as a Source Data file.

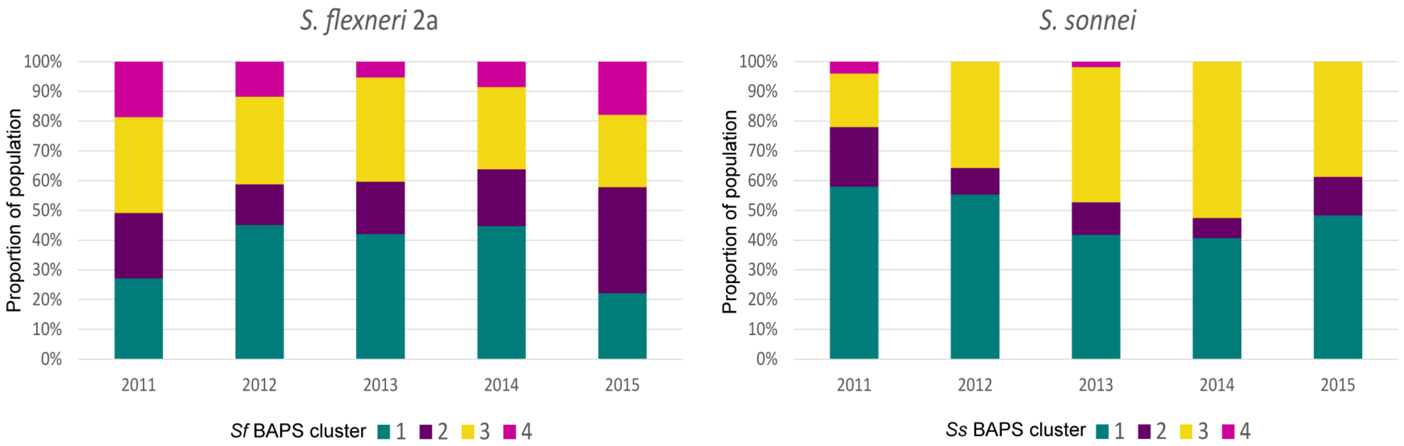

Supplementary figure 4. Relative proportion of sample set by BAPS cluster across study period.

Source data are provided as a Source Data file.
