# Supplementary material for: The genomic epidemiology of shigellosis in South Africa
Source: Nat Commun. 2023 Nov 24;14:7715. doi: 10.1038/s41467-023-43345-5 (PMC10673971; doi:10.1038/s41467-023-43345-5)
Supplement: Supplementary file 3 — Description of Additional Supplementary Files [file 41467_2023_43345_MOESM3_ESM.pdf]

**Description of Additional Supplementary Files**

**File Name:** Supplementary Data 1
**Description:** Supplementary data includes isolate accession numbers and associated metadata, quality control method outputs, phylogenetic reference isolate accession numbers, AMR and virulence genotyping method outputs, gene names and accession numbers for the local virulence gene database, virulence gene association statistics and the virulence loci reference and controls accession numbers
