# Supplementary material for: The genomic epidemiology of shigellosis in South Africa
Source: Nat Commun. 2023 Nov 24;14:7715. doi: 10.1038/s41467-023-43345-5 (PMC10673971; doi:10.1038/s41467-023-43345-5)
Supplement: Supplementary file 5 — Reporting Summary [file 41467_2023_43345_MOESM5_ESM.pdf]

nature portfolio

Corresponding author(s): Prof. Kate Baker and Dr. George Stenhouse

Last updated by author(s): Oct 8, 2023

Reporting Summary

Nature Portfolio wishes to improve the reproducibility of the work that we publish. This form provides structure for consistency and transparency in reporting. For further information on Nature Portfolio policies, see our Editorial Policies and the Editorial Policy Checklist.

Statistics

For all statistical analyses, confirm that the following items are present in the figure legend, table legend, main text, or Methods section.

n/a	Confirmed	
☐	☒	The exact sample size (*n*) for each experimental group/condition, given as a discrete number and unit of measurement
☐	☒	A statement on whether measurements were taken from distinct samples or whether the same sample was measured repeatedly
☐	☒	The statistical test(s) used AND whether they are one- or two-sided<br>*Only common tests should be described solely by name; describe more complex techniques in the Methods section.*
☐	☒	A description of all covariates tested
☐	☒	A description of any assumptions or corrections, such as tests of normality and adjustment for multiple comparisons
☐	☒	A full description of the statistical parameters including central tendency (e.g. means) or other basic estimates (e.g. regression coefficient) AND variation (e.g. standard deviation) or associated estimates of uncertainty (e.g. confidence intervals)
☐	☒	For null hypothesis testing, the test statistic (e.g. *F*, *t*, *r*) with confidence intervals, effect sizes, degrees of freedom and *P* value noted<br>*Give P values as exact values whenever suitable.*
☐	☒	For Bayesian analysis, information on the choice of priors and Markov chain Monte Carlo settings
☐	☒	For hierarchical and complex designs, identification of the appropriate level for tests and full reporting of outcomes
☐	☒	Estimates of effect sizes (e.g. Cohen's *d*, Pearson's *r*), indicating how they were calculated

Our web collection on statistics for biologists contains articles on many of the points above.

Software and code

Policy information about availability of computer code

Data collection	Whole genome sequenced was performed using Illumina HiSeq 4000 sequencing equipment for all isolates. Some isolates (S. flexneri 2a n=119 and S. sonnei n= 65) were re-sequenced with the Illumina NovaSeq 6000 platform.
Data analysis	ShigaTyper (v1.0.6) (https://github.com/CFSAN-Biostatistics/shigatyper) and SonneiTyping sonnei_genotype.py (v1) (https://github.com/katholt/sonneityping). RaxML-NG (v0.6.6; GTR+G substitution model, 1000 bootstrap validation and mid-point rooted). bwa mem (v0.7.17), bcftools (v1.9), Samtools (v1.9), samclip (v2.27.1) and Picard (v2.23.8). phaster, bedtools (v2.27.1). Bayesian Evolutionary Analysis by Sampling Trees software (BEAST2) (v2.6.3), extended bayesian coalescent skyline model, relaxed clock model with log normal prior distribution (prior rate = 1e-6), and BmodelTest site model averaging, transition-transversion split (prior mutation rate = 1.0). Beast2 MCMC chain length was 5043 million steps (sampling every million) for S. flexneri 2a and 3300 million for S. sonnei (sampleing every 0.1 million). Gubbins (v2.4.1). seqmagik (v0.8.0), TempEST v1.5.3). logcombiner, treeannotator (vs.6.3). FigTree (v1.4.4) and ITOL. RhierBAPS (v1.1.3) in Rstudio (r v4.1.0).Unicycler (v0.4.7), Prokka (v1.14.5). StarAMR (v0.5.1)and AMRfinderplus (v3.2.3). VirulenceFinder (v2.0.4-1). Blastn (v2.10.0+). bbmap randomreads (v38.0), seqkit (v0.10.1).

For manuscripts utilizing custom algorithms or software that are central to the research but not yet described in published literature, software must be made available to editors and reviewers. We strongly encourage code deposition in a community repository (e.g. GitHub). See the Nature Portfolio guidelines for submitting code & software for further information.

Data

Policy information about availability of data

All manuscripts must include a data availability statement. This statement should provide the following information, where applicable:

- Accession codes, unique identifiers, or web links for publicly available datasets
- A description of any restrictions on data availability
- For clinical datasets or third party data, please ensure that the statement adheres to our policy

All supporting data have been provided within the article or through supplementary data files. The study isolates' raw sequences have been deposited in the European Nucleotide Archive under project accession PRJEB55173 and individual isolate accession numbers can be found in the Supplementary Data. Accession numbers for reference isolates used in the study are also provided in the supplementary table.

Research involving human participants, their data, or biological material

Policy information about studies with human participants or human data. See also policy information about sex, gender (identity/presentation), and sexual orientation and race, ethnicity and racism.

Reporting on sex and gender	All references to patient hospital recorded sex / gender have been reported as sex
Reporting on race, ethnicity, or other socially relevant groupings	These are not discussed in our study.
Population characteristics	Patients were sampled from according to a standard case of moderate to severe diarrhoea: presenting at a public healthcare facility with a history of diarrhoea defined as three or more loose stools per day, either with or without blood. Patients of all ages and sexes were were included in the study.
Recruitment	Patients were recruited as part of public healthcare surveillance in South Africa. Coverage and access to healthcare is greater in urban areas compared to rural areas, thus the findings of our study may be less relevant in rural areas than urban areas. Sampling was also conducted at public hospitals, which likely have a different patient demographic than private healthcare facilities in the region. Thus it is possible that our study was unable to detect shigellosis associated with certain demographics, such as the community of men who have sex with men, we found no phylogenetic signal suggestive of transmission within this community but this is unlikely to mean transmission is not occurring. Similarly, requiring patients to attend hospital means that less symptomatic patients will not be sampled from. All together, through these sampling methods we will have under-sampled from some human sub-populations and potential some Shigella sub-populations, and through this perhaps have missed important epidemiological factors affecting shigellosis in South Africa
Ethics oversight	No individual consent was required or sought as all isolates were collected as part of routine surveillance and ethical approval for the use of patient data for public health activities was granted by the Human Research Ethics Committee of the University of the Witwatersrand (Protocol Numbers: M060449 and M110499).

Note that full information on the approval of the study protocol must also be provided in the manuscript.

Field-specific reporting

Please select the one below that is the best fit for your research. If you are not sure, read the appropriate sections before making your selection.

☒ Life sciences ☐ Behavioural & social sciences ☐ Ecological, evolutionary & environmental sciences

For a reference copy of the document with all sections, see nature.com/documents/nr-reporting-summary-flat.pdf

Life sciences study design

All studies must disclose on these points even when the disclosure is negative.

Sample size	No sample size calculation was performed as this was a descriptive pilot study. However, sample sizes were comparable to similar initial national studies describing pathogen genomic epidemiology.
Data exclusions	Data excluded from some statistical analyses where necessary metadata unknown. Additionally, four isolates were excluded from the BEAST2 analyses due to being outliers in molecular clock signal, visually assessed in TempEst(v.1.5.3)
Replication	Used publicly available software and standard statistical methods for analyses, recording all non-default parameters/settings.
Randomization	Sub-sampling of isolates from the larger surveillance dataset was randomised. Random sub-sampling was achieved by selecting every 8th (S. flexneri 2a) or 5th (S. sonnei) isolate, based on SA lab number, from the database of collected, surveillance isolates (collected from any body site, 1 January 2011 – 31 December 2015, for which demographic data was available). Associated patient metadata was used in further analyses and was also, therefore, randomized through initial random selection.
Blinding	Blinding is not relevant for our study as no interventions were given.

Reporting for specific materials, systems and methods

We require information from authors about some types of materials, experimental systems and methods used in many studies. Here, indicate whether each material, system or method listed is relevant to your study. If you are not sure if a list item applies to your research, read the appropriate section before selecting a response.

Materials & experimental systems

n/a	Involved in the study
☒	☐ Antibodies
☒	☐ Eukaryotic cell lines
☒	☐ Palaeontology and archaeology
☒	☐ Animals and other organisms
☒	☐ Clinical data
☒	☐ Dual use research of concern
☒	☐ Plants

Methods

n/a	Involved in the study
☒	☐ ChIP-seq
☒	☐ Flow cytometry
☒	☐ MRI-based neuroimaging

Plants

Seed stocks	*Report on the source of all seed stocks or other plant material used. If applicable, state the seed stock centre and catalogue number. If plant specimens were collected from the field, describe the collection location, date and sampling procedures.*
Novel plant genotypes	*Describe the methods by which all novel plant genotypes were produced. This includes those generated by transgenic approaches, gene editing, chemical/radiation-based mutagenesis and hybridization. For transgenic lines, describe the transformation method, the number of independent lines analyzed and the generation upon which experiments were performed. For gene-edited lines, describe the editor used, the endogenous sequence targeted for editing, the targeting guide RNA sequence (if applicable) and how the editor was applied.*
Authentication	*Describe any authentication procedures for each seed stock used or novel genotype generated. Describe any experiments used to assess the effect of a mutation and, where applicable, how potential secondary effects (e.g. second site T-DNA insertions, mosiacism, off-target gene editing) were examined.*
